# Compliance to the Integrated School Health Policy: Intersectoral and multisectoral collaboration

**DOI:** 10.4102/curationis.v42i1.1912

**Published:** 2019-02-25

**Authors:** Richard M. Rasesemola, Gert P. Matshoge, Tendani S. Ramukumba

**Affiliations:** 1 Adelaide Tambo School of Nursing Science, Faculty of Science, Tshwane University of Technology, South Africa

## Abstract

**Background:**

Implementation of the Integrated School Health Policy (ISHP) requires strong intersectoral collaboration on the part of key role players such as the Department of Health, Department of Basic Education and Department of Social Development. These departments and educational structures such as school governing bodies, teacher unions and learner organisations, academic institutions, civil society and development partner organisations are also expected to contribute to the development of sustainable and comprehensive school health programmes.

**Objectives:**

The objective of this study was to describe the compliance of the schools in the City of Tshwane to the ISHP in 2015.

**Method:**

A quantitative, explorative and descriptive study was conducted in the City of Tshwane using a questionnaire to determine the extent of compliance to the application of the ISHP in selected schools.

**Results:**

The results indicated a widespread non-compliance to ISHP programmes. There was insufficient stakeholder integration in the school health programmes at schools in the City of Tshwane.

**Conclusion:**

The lack of collaboration with relevant stakeholders in school health service delivery will lead to a fragmented, uncoordinated and unsustainable approach to the execution of ISHP programmes. This might result in delayed or no detection and intervention in cases of, among others, mental, psychosocial and health challenges to learning, as well as development of nutrition-related conditions.

## Introduction

A multidisciplinary and collaborative healthcare approach has been fundamental to the World Health Organization’s (WHO) constitutional foundation for its health and social development agencies (WHO 1998a). Subsequently, the WHO established a number of school health programmes within its agencies, such as the Global School Health Initiative, which has been vital in advocacy for school health policies and programmes in many countries.

In 2012, the South African government initiated the Integrated School Health Policy (ISHP), a policy initiative that is aimed at improving the health of schoolgoing children and their respective communities (Department of Health [DOH] & Department of Basic Education [DOBE] 2012). The policy’s objective, as announced by the president of South Africa at the time, was to serve as a guideline for the provision of school health services just as has been done in other African countries such as Botswana, Ghana and Nigeria. The policy initially required two key departments, namely the DOH and DOBE, to collaborate in the provision of integrated school health services. Furthermore, ISHP according to *Health-e News* (2013) aims to:

provide a more comprehensive package of services, which addresses not only barriers to learning, but also other conditions which contribute to morbidity and mortality amongst learners during both childhood and adulthood. The programme also includes a new, more prominent emphasis on the provision of health services in schools, which previously only conducted health screenings and referrals.

Other activities that should make up the package that should be provided in all schools include learner participation, community participation, coordination and partnership, and health education and promotion (DOH & DOBE 2012:12–16).

According to the DOH and DOBE (2012:17), implementing the ISHP requires a commitment to close collaboration between all role players, with the DOH, DOBE and now the Department of Social Development as well taking joint responsibility for ensuring that the ISHP is comprehensive and sustainable. It is for this reason that the revised policy was co-signed by the Ministers of Health and Basic Education. The ISHP’s preamble stresses the commitment of all other role players to close collaboration to ensure that the objectives of the policy are fully achieved. The policy document contains a list of coordination and partnership proposals that includes community role players in school health promotion as a key strategic objective (DOH & DOBE 2012).

The implementation of school health programmes has been a challenge in South Africa, as in other sub-Saharan countries. Botswana, Ghana and Nigeria have had more than a decade of comprehensive school health policies and programmes running, and non-compliance to the WHO’s recommended minimum standards has been the central concern. Non-compliance has been noted in areas such as school-based reproductive health, physical examination and record-keeping in the Nigerian school health system (Akpabio 2010:19; Ojugo 2005:2), while confidentiality during physical assessments and environmental care awareness in schools have been identified as challenge areas in Botswana’s school health system (Shaibu & Phaladze 2010:199). Fragmentation of school health services, as identified by the director of basic education, was the major reason for non-compliance to the school health policy in Ghana (Ghana News Agency 2014).

From the literature, it is evident that certain regions and developed countries have advanced in the WHO school health programmes and comply with the minimum standards for their respective regions, such as the Americas, Europe, the West Pacific region and the South-East Asia region (Birdthistle & WHO 2000). The authors have noted with concern that, in South Africa, basic school health services are not offered constantly, consistently and systematically. Some children complete school without ever being seen by a school health nurse. The state of infrastructure and the surrounding environment of some schools around the City of Tshwane (COT) appears dilapidated. The environment has the potential to bring about violent and harmful activities among learners and the surrounding communities, a situation that has led to unfavourable reports in the media. Such reports include the surge of violent activities in schools around the country such as the assault of a teacher in Limpopo province by a Grade 12 pupil and the murder of a teacher by a 17-year-old pupil at a school in North West province (Modise 2018).

According to Spotlight (2013), the ISHP recommends that learners should take part in the implementation of health policy in their schools and communities. The policy acknowledges international evidence indicating that successful school health programmes are based on strong partnerships between education and health sectors, teachers and health workers, schools and community groups, students and people responsible for school health programmes (Spotlight 2013). The ISHP also recognises the need for collaboration between the national and provincial DOH, DOBE and Department of Social Development to supervise collaboration between all parties taking part in the programme (Spotlight 2013).

The question regarding the schools’ compliance with the ISHP, a policy guideline for both the DOH and the DOBE, necessitated the development of this study. The purpose of the study was to explore and describe the compliance with the ISHP by schools in the COT across all quintile classifications, with the objective of informing the guidelines for compliance to the school health policy in the COT.

## Research methods and design

### Study design

A quantitative, explorative, descriptive and cross-sectional survey was conducted in selected schools in the COT, using a questionnaire to determine the extent of compliance to the application of the ISHP.

### Setting

The study was conducted in the COT, which is the second largest municipality in Gauteng province, is among the six biggest metropolitan cities in the country (COT 2015) and is the administrative capital of South Africa (Worldatlas 2017). It is home to 598 public schools from quintiles 1 to 5 (see Table 1), accommodating 448 720 learners and 16 258 teachers, including 25 schools in the special schools category that cater for children with disabilities (Gauteng Department of Education n.d.).

**TABLE 1:** City of Tshwane school categories indicating population and targeted sample.

Classification	Number of schools	Targeted sample
Quintiles 1 and 2	149	44
Quintile 3	77	23
Quintile 4	113	40
Quintile 5	122	36
Special schools	25	7
Unclassified schools	112	36

**Total**	**598**	**179**

### Study population and sampling strategy

All public schools in the COT were eligible for inclusion in the study and accessible in terms of the DOBE’s permission. The schools formed the units of analysis. Table 1 shows the categorisation of schools and the number of each category sampled. According to Collingridge (2013), the quintile system allocates all government schools into one of five categories, with quintiles 1 and 2 schools designating the poorest institutions while quintile 5 denotes the least poor public schools. Collingridge (2013) further states that the quintile to which a school is assigned is based on the rates of income, unemployment and illiteracy within the school’s catchment area. Although in 2013 the South African government wanted to do away with the quintile system, the Minister of Basic Education mentioned that it would be retained to help inform the department on aspects such as post provisioning, possible performance awards for schools and other programmes such as school nutrition and transport (Collingridge 2013). Table 1 depicts school categories indicating population and targeted sample.

Stratified convenient sampling was applied in this study to select and stratify the sample according to quintile classification. On the advice of a statistician, convenient sampling of 30% of each quintile classification was performed to ensure representation and generalisability of results (Burns & Grove 2005:348). The sample was based on the targeted population of 598 schools in the COT. School principals were elected to be participants on behalf of the schools. In cases where principals were not available, their deputies were elected. The principals or their deputies were chosen because, according to the *South African Schools Act* (Government of South Africa 1996), they are appointed to manage schools, thus becoming the custodians of the schools they manage.

In total, 179 schools were sampled and questionnaires were hand-distributed to be completed by the school principals or their deputies. However, because of Lasagna’s law, which holds that availability of research subjects is often overestimated because most respondents would either not return the questionnaires or would simply not be available (Van der Wouden et al. 2007), only 66 schools responded – a 36.87% response rate. In an effort to try and reach the projected sample size, follow-ups were done via telephonic reminders and physical visits to the principals who had not returned the questionnaires. Those principals who did not comprehend the contents of the questionnaire were assisted in the completion of the questionnaire, while some exercised the right to withdraw their participation from the study. This drastically reduced the projected target population.

### Data collection

Data were collected over a period of 6 months, from August 2015 to February 2016. The principals or their deputies responded to series of questions in a questionnaire. The questionnaire contained the 13 elements of the ISHP; however, only one element of multidisciplinary and multisectoral collaboration was presented in this article. The development of the questionnaire was guided by the principles of the ISHP and WHO principles of school health systems.

### Data analysis

Data analysis included data cleaning and capturing. Descriptive statistical analysis was performed through the use of Excel; the interpretations were presented as frequencies and proportions as expressed in percentages.

### Validity and reliability

The content used to select the questions in the questionnaire was guided by ISHP document. Three specialists in the field of school health then checked the questionnaire before it was presented to the multidepartmental review committee for peer review. Further, the questionnaire was pretested by collecting data at three schools that did not form part of the main study. The reliability of the measuring instrument was consistent with proven methodologies applied in social or humanity studies and as such can be replicated in similar studies across the school health population elsewhere in the country (Brink, Van der Walt & Van Rensburg 2012:126).

### Ethical consideration

This study conformed to moral, ethical and legal standards of scientific inquiry, including ethical clearance by the Committee of Departmental Research and Innovation, Tshwane University of Technology Research Ethics Committee, Faculty Higher Degrees Committee, and Faculty Research Ethics Committee (FCRE 2015/04/003 [02] [SCI]). Permission was obtained from the Department of Basic Education regional office and the district directorate in Gauteng province (D2016/206), and consent was obtained from the schools’ principals or their deputies on behalf of their schools. The principals’ names and the names of schools were not included in any reports to ensure privacy and confidentiality.

## Results

Regarding the presence of intersectoral and multidisciplinary collaboration between schools and other stakeholders to ensure compliance to the ISHP, non-compliance in schools around the COT was indicated as depicted in Table 2. For the purpose of this article, sufficient compliance to the ISHP’s elements of multidisciplinary and multisectoral collaboration refers to a minimum of 75% of schools having indicated the presence of those elements in their schools.

**TABLE 2:** Integrated School Health Policy (ISHP) multidisciplinary and multisectoral collaboration (*N* = 66).

ISHP collaboration elements	Yes		No		Total (%)
	Percentage (%)	Frequency (*n*)	Percentage (%)	Frequency (*n*)	
Involvement of parents or families of learners	30	20	70	46	100
Involvement of community members	30	20	70	46	100
Involvement of local health departments, agencies or organisations	58	38	42	28	100
Involvement of faith-based organisations	60	40	40	26	100
Involvement of learner bodies (learner representative committee)	14	9	86	57	100
Involvement of physical education staff	62	41	38	25	100
Involvement of mental health or social services staff	14	9	86	57	100
Involvement of nutrition or food service staff	52	34	48	32	100
Involvement of health services staff (e.g. school nurses)	35	23	65	43	100
Involvement of transportation staff	35	23	65	43	100

### Involvement of parents or families of learners

It appears from Table 2 that the majority of the studied schools in the COT did not comply with broader community collaboration compliance requirements set out in the ISHP guidelines. The results showed schools’ non-compliance to parental or family integration in terms of their integration in collaborative activities and roles in matters of school health services at 70% (*n* = 46).

### Involvement of school health community members

Integration of community members in matters of school heath was deficient in schools in the COT at 70% (*n* = 46). This means that the communities within which schools are found do not necessarily form part of the school health service delivery system.

### Involvement of school-based local health departments, agencies or organisations

School health services should form part of the reengineered primary healthcare services stipulated in the National Health Insurance Policy (DOH 2017:29). Furthermore, the ISHP highlights the need to integrate local health agencies such as primary healthcare facilities into the school health services (DOH & DOBE 2012). However, the results indicated that 58% (*n* = 38) of studied schools in the COT did not sufficiently collaborate with local health departments, agencies or organisations to meet the comprehensive healthcare requirements.

### Involvement of faith-based organisations

The ISHP also quotes the element of involving faith-based organisations in school health delivery. According to the DOH and DOBE (2012:16), community structures play an important role for improved health of learners in schools. Active involvement of school governing bodies, community leaders such as traditional and faith-based leaders and ward councillors, as well as the buy-in of the entire school community, is required for the success of the ISHP. However, 60% (*n* = 40) of schools in the COT did not have sufficient compliance with regard to the involvement of faith-based organisations.

### Involvement of school-based learner bodies

According to the DOH and DOBE (2012:16), participation of learners through student representative councils and other organisations will further ensure successful implementation of the ISHP. However, participants indicated that 86% (*n* = 57) of studied schools in the COT did not integrate learner representative bodies into the school health services to ensure the ISHP’s successful implementation through platforms created at schools and community level.

### Involvement of school-based physical education staff

Physical education was officially dropped as a subject in South Africa in 1994, partly because of crowded curriculum, loss of time allocation, lower perceived status of physical education in general, and insufficient financial and material resources (Du Toit, Van der Merwe & Rossouw 2007:241). In the current study, 62% (*n* = 41) of respondents reported that they had physical education staff. This means that some schools in the COT still have physical education included in their curriculum and show compliance to the ISHP.

### Involvement of school-based mental health and social services

According to the DOH and DOBE (2012:25–26), local literature indicates high prevalence rates for anxiety disorders, post-traumatic stress disorders, depression and conduct disorders among children and adolescents. Several factors, such as biological, social and psychological factors, are known to contribute to the high prevalence of mental disorders among young people, while poor mental health is associated with such effects as educational underachievement, social disadvantage, and poor health and well-being. The mental health needs of children and adolescents can be addressed at numerous levels and intervention sites, and schools can play an important role. At a rate of 86% (*n* = 57), schools in the COT indicated that they did not have collaboration with or integrate mental health or social development services into school health services.

### Involvement of school-based nutrition and food services

In 2000, the World Education Forum held in Dakar made a strong case that provision of effective school health services is an important strategy for achieving education for all (DOH & DOBE 2012:6). The forum further recommended that school-based health and nutrition services be provided together in all schools as the basic components of a school health programme. The Commission on the Social Determinants of Health has also called for greater investment in comprehensive early childhood development that links families and young children to health, education and nutrition services (DOH & DOBE 2012). In this study, the results indicated that 52% (*n* = 34) of schools in the COT did not collaborate or integrate nutrition or food service staff.

### Involvement of school health services

Implementation of integrated school health programmes forms part of the four main streams of key health reform of primary healthcare reengineering (DOH & DOBE 2012:29). School health services are being provided to improve the physical, mental and general well-being of children of schoolgoing age. The ISHP provides a range of promotive, preventive and curative services and includes a focus on screening for health-related barriers to learning such as vision, hearing, mental health, and cognitive and related developmental impairment (DOH & DOBE 2012:30). According to the DOH and DOBE (2012:21), the school health team should be led by a professional nurse. However, the results from the schools in the COT indicated that 65% (*n* = 43) of the schools did not have any collaboration or integrate school health nurses in their school health services.

### Involvement of scholar transportation

In the process of delivering the curriculum, the Gauteng Department of Education learned that some learners are deprived of access to public schools because of the distance they have to travel to get to school (Gauteng Department of Education n.d.:5). Scholar transport is a necessary and integral part of the right to basic education and learners who cannot get transport suffer (Joseph & Carpenter n.d.:276). Therefore, it is the responsibility of the school principal together with the school management team to identify those learners whose learning is interrupted because of the distance they have to travel between home and school (Gauteng Department of Education n.d.:7). From Table 2, the results showed that 65% (*n* = 43) of the participants indicated that there was no integration of transportation staff into their school health services.

## Discussion

According to the International Union for Health Promotion and Education (2009, cited by Clelland, Cushman & Hawkins 2013:1), the Health Promoting School (HPS) initiative maintains that, in order to successfully promote the long-term health and well-being of learners, schools must work closely with parents and the local community. In her research, Kwatubana (2014:1458) argues that ‘full, meaningful community participation completes any model of health promotion implemented in any schools’.

Community participation is referred to as both the processes and activities that allow members of a particular population to be heard, empowering them to be part of decision-making processes and enabling them to take action in conjunction with other structures on health promotion issues (Inter-Agency Network for Education in Emergencies 2004:80, cited by Kwatubana 2014:1458).

The results in this study indicated COT schools’ lack of collaboration with parents and families of learners, as well as community members, posing poor compliance to the implementation of ISHP programmes. According to the Centers for Disease Control and Prevention (CDC 2012:7), the engagement of learners’ parents and families in school health services promotes positive health behaviours among learners. The CDC (2012:1) further adds that parent engagement in schools contributes to learners’ lifelong health and learning. Literature has also confirmed that parental collaboration in school health services is more likely to bring about positive outcomes in learners, such as higher grades and test scores, better learner conduct and improved social skills (CDC 2012:1). The same learners are less likely to be involved in harmful sociocultural activities such as cigarette smoking, alcohol consumption, becoming pregnant, physical inactivity and emotional distress (CDC 2012:1).

Health promotion in schools, according to Clelland et al. (2013:6), will only be effective if schools ensure that all components of the HPS approach are working in collaboration to effectively enhance learners’ well-being. The CDC has suggested some strategies and sample actions teachers can take to increase parent engagement in school health services. Such strategies include schools making an active, deliberate and positive connection with the parents of the learners, providing a variety of formal opportunities to engage parents in school health activities and working with parents to sustain parent engagement in school health services (CDC 2012:2–3).

However, Clelland et al. (2013:6) mention that key challenges to encourage parental involvement exist, particularly the issue of whose role it is to educate parents so that they are equipped to support their children in learning about health. Teachers consider learners’ educational outcomes as their primary responsibility; therefore, educating parents is beyond the scope of school services (Clelland et al. 2013:6). The status in COT schools indicates non-compliance to the integration and collaboration with the communities within which the schools are found and learners reside. This is despite the fact that the ISHP document outlines that advocacy and social mobilisation should be conducted in collaboration with essential role players at national, provincial and district levels in all government departments, other health programmes, NGOs rendering health services to the school community, school governing bodies (SGBs), parents and learners (DOH & DOBE 2012:22).

Through its Global School Health Programme, the WHO has been fostering the concept of HPSs since 1995 (DOH & DOBE 2012:29). The HPS framework utilises health promotion concepts and incorporates three collaborative elements: curriculum learning and teaching, the ethos and environment of a school, and links with parents and the wider community (Clelland et al. 2013:1). The national DOH (2000, cited by DOH & DOBE 2012:29–30) alludes that HPSs are schools that:

- engage health and education officials, educators, pupils, parents and community leaders in efforts to promote health
- strive to provide a healthy environment, school health education and teaching, school health services and school and community projects and outreach
- strive to improve the health of school personnel, families and community members, as well as pupils, and work with community leaders to help them understand how the community contributes to, helps or undermines health and education.

To achieve the WHO’s HPS objective through the ISHP, there is a need for collaboration between the parents or families of the learners and community within which the schools are found. The CDC (2014:5) suggests that schools and communities collaborate in aligning resources in support of the whole child.

According to the DOH and DOBE (2012:16), the participation of learners in physical school health programmes in collaboration with student representative councils and other organisations will ensure successful implementation of the ISHP. This means that learner bodies need to be consulted and encouraged to support the implementation of the ISHP through platforms created at school and community level. Willms (2003:8) states that school is essential to the daily life of many youths. It is viewed as essential to their lifelong well-being, and this attitude should be reflected in their participation in academic and non-academic school programmes. Willms (2003:8) further mentions that these learners tend to foster good relations with school staff and other learners and have a sense of belonging to the school. The current study indicated schools’ non-compliance with the integration and collaboration with learner bodies in school health service delivery. This might result in a lack of sense of belonging and poor confidence in academic success among learners, which may have a strong bearing on their future and may result in dissatisfaction with school (Willms 2003:8).

The ISHP requires school health to be designated at a school and school health becomes the nurse’s primary and core responsibility, rather than an add-on to other duties (DOH & DOBE 2012:9–10). The status quo in COT schools at the moment is characterised by lack of integration and collaboration with local health departments, agencies or organisations, mental health and social services staff, and health service staff in school health services, which is a recipe for non-compliance to the ISHP. According to the DOH and DOBE (2012:9–10), school health services are currently delivered by designated school health nurses, who form part of the primary healthcare staff component. Both departments have identified a number of challenges that limit schools’ compliance to the ISHP, such as insufficient staff and infrequent visits by the school health nurse to schools, lack of resources, mental health assessment not being done because of lack of facilities and resources, and referral systems not being available to respond to the identified health needs. The recommended norm for delivering individual learner assessments according to the DOH and DOBE (2012:21) is one professional nurse for every 2000 learners to be assessed per year. The sole professional nurse is primarily responsible for coordinating the implementation of the ISHP and conducting individual learner assessments on site.

School nutritional services form part of the ISHP school health package from Grade R to Grade 12 (DOH & DOBE 2012:14), and school feeding scheme programmes form one of the most important components of the value chain in the school health system (WHO 2012). In the 2013–2014 financial year, the South African government allocated R5.2 billion in conditional grant transfers to provinces for the National School Nutrition Programme (Republic of South Africa National Treasury 2015, cited by Graham et al. 2015:9), a nutritional intervention programme that provides nutrition to 8.8 million poor children in schools in the nine provinces (DOBE 2014). According to Swart (2009, cited by Graham et al. 2015:10), feeding schemes have been in existence in South Africa since 1916, when they only benefitted the white population, although they were supposed to benefit poor children. Graham et al. (2015:10) further mention that child hunger because of inadequate nutrition intake among black communities was severe. This may be a result of poor collaboration between all stakeholders.

According to Agüero, Carter and Woolard (2006), childhood nutritional deprivation can have severe and long-lasting negative effects on children’s physical and intellectual development, while the Integrated Nutrition Programme highlights the essential role played by nutrition for survival, sustained health, growth, mental and physical development, performance and overall academic productivity from childhood into adulthood (DOH & DOBE 2012:33). Therefore, collaborative nutritional interventions should be integrated into all aspects of a school community’s physical and psychosocial life and should as such be integrated into food and feeding programmes, physical exercise, sports and recreational activities (WHO 1998b). These programmes need to be well-structured to improve and maintain the value in the overall health of the child in school and the community in which they live (WHO 2012). They also need to be well balanced to include, among others, a nurse, dietician, social workers and food safety.

The WHO’s Expert Committee on Comprehensive School Health Education and Promotion outlines best practice guidelines for integrated school nutrition programmes (WHO 1997). The committee suggests that the point of departure in the integration process in developing countries would be to assess protein-energy malnutrition, micronutrient deficiencies and short-term hunger, as well as paediatric body mass index screening (Pietras et al. 2012:107–108). This approach will therefore integrate nutrition programmes into feeding schemes, nutritional education and teaching, micronutrient supplementation programmes and school-based deworming programmes. The focus areas addressed within this programme include household and food security, growth monitoring and promotion, control of micronutrient deficiencies, nutrition education, disease-specific nutrition support and service management. The school nutrition programme therefore forms an important pillar of the Integrated Nutrition Programme. However, the results in Table 2 reflected non-compliance to integrated and collaborated food services in the COT schools, which consequently threatens compliance to the ISHP. Having very few or no visitations from school health nurses also deprives learners of a much-needed assessment of nutrition-related conditions by the school health nurse.

The 2005 National Food Consumption Survey found that 18% of children were stunted, 9.3% were underweight and 4.5% were wasted, while 20% were overweight and 5.34% were obese (DOH & DOBE 2012:23). Nutrition-related conditions in learners, such as obesity, has prompted research and development of school nutrition programme elements such as control of nutritional value of food items accessible by or sold to schoolchildren across the globe (Snelling & Yezek 2012:91–92).

By 2013, percentages of schoolchildren walking long distances to school had risen in South Africa (Grant 2017). Scholars in poor, rural and lower quintile schools are the most affected as the statistics show that more than 80% of learners from quintiles 1 and 2 schools walk to school on a daily basis.

The Gauteng Department of Education (2011) approved a scholar transport policy after it was established that some learners are deprived of the right to education because of long distances travelled between their homes and schools. The policy was established to provide best practice guidelines for the provision of scholar transportation services for learners to ensure that they are punctual, arrive safely at schools and that their learning is not interrupted (Gauteng Department of Education 2011). The policy also mentions that it is the principal’s responsibility to identify those learners whose education is interrupted because of the long distances they travel to and from school.

From the results in Table 2, it has been established that most schools in the COT do not have collaboration with the scholar transportation services. According to Joseph and Carpenter (n.d.:276), children who walk long distances to school face very real and ever-present dangers of crime, while safe and reliable scholar transport would allow them to be protected from crime and give them much-needed peace of mind. Even those scholars who use public transport such as taxis and buses to get to schools are not free from dangers of exhaustion and road accidents. Seven per cent of scholars from quintiles 3 and 4 travel to school by public buses and around 20% of scholars from quintiles 4 and 5 get to school by minibus taxis (Macupe 2017).

Media reports have on multiple occasions reported on road accidents involving large numbers of schoolchildren. According to Macupe (2017), 27 pupils died in car crashes between January and August 2017 travelling to or from school. Macupe (2017) further states that these are numbers that have been reported but the real statistics could be much higher. Most of these accidents are reported as having been primarily being attributed to mechanical faults of the vehicles. However, Ramukumba and Mathikhi (2016) also established that taxi drivers’ health goes unchecked because of the pressure that comes with the nature of their work and some are at risk of diseases such as cardiovascular diseases, diabetes and its complications, and other chronic diseases.

## Strengths and limitations

The data collection instrument was too long and time-consuming, and some principals withdrew their participation. The results of the study could not be generalised because of poor participation (return rate of 36.87%). Lastly, the research tool did not include the quintile classification of schools to allow the researcher to perform inferential analysis and assessment of distribution of resources.

## Recommendations

Schools’ missions and visions should reflect on their intention to collaborate with other stakeholders. Teachers need to be trained on their roles in the implementation of ISHP programmes and their roles need to be clarified in the ISHP document. Intersectoral and multisectoral collaboration for implementation of ISHP programmes should be strengthened with explicit roles and participation of all stakeholders in the ISHP programmes clearly outlined in the ISHP document. As institutions in their own right, schools have to establish baseline standards of compliance to the ISHP that would be used as core standards to sustain and accredit them. Curriculum development for integrated school health promotion and teaching implementation, as well as execution and development of an integrated school health toolkit to guide the implementation process should be done as a matter of urgency. Lastly, there is a need to establish a policy oversight authority to oversee schools’ compliance to ISHP programmes.

## Conclusion

There is widespread non-compliance to integration and collaboration with different stakeholders for the delivery of school health services as stipulated in the ISHP. This may affect schoolgoing children in many different ways. The fragmented, uncoordinated and unsustainable approach to the execution of ISHP programmes will lead to the alienation of parents and communities in school health and welfare participation and children’s learning. It will also lead to children finishing school without being seen by a school health nurse for early detection and intervention in cases of, among others, mental, psychosocial and health challenges to learning, and development of nutrition-related conditions.

## References

[CIT0001] Agüero J.M., Carter M.R. & Woolard I., 2006, *The impact of unconditional cash transfers on nutrition: The South African Child Support Grant*, Southern Africa Labour and Development Research Unit working paper 6/8, viewed 13 July 2018, from http://opensaldru.uct.ac.za/bitstream/handle/11090/46/06_08.pdf?sequence=1

[CIT0002] Akpabio I., 2010, ‘Problems and challenges of school health nursing in Akwa Ibom and Cross River states, Nigeria’, *Continental Journal of Nursing Science* 2, 17–28.

[CIT0003] Birdthistle I. & World Health Organization, 2000, *Improving health through schools: National and international strategies*, World Health Organization, Geneva.

[CIT0004] Brink H., Van der Walt C. & Van Rensburg G., 2012, *Fundamentals of research methodology for health care professionals*, 3rd ed, Juta, Cape Town.

[CIT0005] Burns N. & Grove S.K., 2005, *The practice of nursing research: Conduct, critique and utilization*, 5th edn., Elsevier/Saunders, Philadelphia, PA.

[CIT0006] Centers for Disease Control and Prevention, 2012, *Parent engagement: Strategies for involving parents in school health*, viewed 13 July 2018 from https://www.cdc.gov/healthyyouth/protective/pdf/parent_engagement_strategies.pdf

[CIT0007] Centers for Disease Control and Prevention, 2014, *Promoting parent engagement: Improving student health and academic achievement*, viewed 13 July 2018, from www.cdc.gov/healthyyouth/protective/pdf/parentengagement_teachers.pdf

[CIT0008] City of Tshwane, 2015, *About City of Tshwane*, viewed 13 July 2018, from http://www.tshwane.gov.za/sites/about_tshwane/Pages/About-Tshwane.aspx

[CIT0009] Clelland T., Cushman P. & Hawkins J., 2013, ‘Challenges of parental involvement within a health promoting school framework in New Zealand’, *Education Research International* 2013, article ID 131636, viewed 13 July 2018, from https://www.hindawi.com/journals/edri/2013/131636/

[CIT0010] Collingridge L., 2013, ‘Schools quintile system to change?’, *Corruption Watch*, viewed 13 July 2018, from http://www.corruptionwatch.org.za/schools-quintile-system-to-change/

[CIT0011] Department of Basic Education, 2014, *National school nutrition programme grants framework 2014/2015*, Department of Basic Education, Pretoria.

[CIT0012] Department of Health, 2017, *National health insurance policy*, Government Printers, Pretoria.

[CIT0013] Departments of Health and Basic Education, 2012, *Integrated school health policy*, Government Printers, Pretoria, viewed 04 April 2017, from http://www.hst.org.za/sites/default/files/Integrated_School_Health_Policy.pdf

[CIT0014] Du Toit D., Van Der Merwe N. & Rossouw J.P., 2007, ‘Return of physical education to the curriculum: Problems and challenges facing schools in South African communities’, *African Journal For Physical, Health Education, Recreation and Dance* 13(3), 241–253.

[CIT0015] Gauteng Department of Education, 2011, *Scholar transport policy*, Gauteng Department of Education, Pretoria.

[CIT0016] Gauteng Department of Education, n.d., *Schools*, viewed 19 April 2017, from http://www.education.gpg.gov.za/Schools/Pages/default.aspx

[CIT0017] Ghana News Agency, 2014, *GES launches school health education programme policy guidelines*, viewed 13 July 2018, from http://www.ghananewsagency.org/education/ges-launches-school-health-education-programme-policy-guidelines-70588

[CIT0018] Government of South Africa, 1996, *South African Schools Act, No. 84 of 1996*, Government Printers, Pretoria, viewed 13 July 2018, from https://www.education.gov.za/LinkClick.aspx?fileticket=aIolZ6UsZ5U%3d&tabid=185&mid=1828

[CIT0019] Graham L., Hochfeld T., Stuart L. & Van Gent M., 2015, *Evaluation study of the national school nutrition programme and the Tiger Brands Foundation in-school breakfast programme in the Lady Frere and Qumbu districts of the Eastern Cape*, Centre for Social Development in Africa, University of Johannesburg, Johannesburg.

[CIT0020] Grant L., 2014, ‘How do South Africa’s children travel to school?’, *Mail & Guardian*, viewed 13 July 2018, from https://mg.co.za/data/2014-07-22-how-do-south-africas-children-travel-to-school

[CIT0021] Joseph S. & Carpenter J. n.d., ‘Scholar transport’, in *Education rights handbook*, viewed 13 July 2018, from https://eelawcentre.org.za/wp-content/uploads/2017/03/chapter-16-copy.pdf

[CIT0022] Health-e News, 2013, ‘Policy: South African Integrated School Health Programme’, *Health-e News*, viewed 02 October 2018, from https://www.health-e.org.za/2013/10/24/integrated-school-health-policy/

[CIT0023] Kwatubana S.J., 2014, ‘School community participation and school health promotion: Challenges and opportunities’, *Mediterranean Journal of Social Sciences* 5(27), 1458–1466, viewed 13 July 2018, from http://www.mcser.org/journal/index.php/mjss/article/view/5230/5047

[CIT0024] Macupe B., 2017, ‘Pupils are condemned to death road’, *Mail & Guardian*, viewed 13 July 2018, from https://mg.co.za/article/2017-08-25-00-pupils-are-condemned-to-death-road

[CIT0025] Modise K., 2018, ‘Learner violence: Talks with families, pupils needed to find solutions’, *Eye Witness News*, viewed 02 October 2018, from https://ewn.co.za/2018/09/21/learner-violence-talks-with-families-pupils-needed-to-find-solutions

[CIT0026] Ojugo A.I., 2005, ‘Status of health appraisal services for primary school children in Edo state, Nigeria’, *International Electronic Journal of Health Education* 8, 146–152, viewed 13 July 2018, from http://files.eric.ed.gov/fulltext/EJ794080.pdf

[CIT0027] Pietras S.A., Rhodes E.T., Meyers A. & Goodman E., 2012, ‘Understanding paediatricians’ views toward school-based BMI screening in Massachusetts: A pilot study’, *Journal of School Health* 82(3), 107–114. 10.1111/j.1746-1561.2011.00673.x 22320334

[CIT0028] Ramukumba T.S. & Mathikhi M.S., 2016, ‘Health assessment of taxi drivers in the City of Tshwane’, *Curationis* 39(1), a1671, viewed 13 July 2018, from https://curationis.org.za/index.php/curationis/article/viewFile/1671/2067 10.4102/curationis.v39i1.1671 PMC6091643 28155302

[CIT0029] Shaibu S. & Phaladze N.A., 2010, ‘School health: The challenges to service delivery in Botswana’, *Primary Health Care Research and Development* 11(2), 197–202. 10.1017/S1463423609990417

[CIT0030] Snelling A.M. & Yezek J., 2012, ‘The effect of nutrient-based standards on competitive foods in three schools: Potential savings in kilocalories and grams of fat’, *Journal of School Health* 82(2), 91–96. 10.1111/j.1746-1561.2011.00671.x 22239134

[CIT0031] Spotlight, 2013, *ISHP analysed*, viewed 13 July 2018, from https://www.spotlightnsp.co.za/2013/06/07/ishp-analysed/

[CIT0032] Van Der Wouden J.C., Blankenstein A.H., Huibers M.J.H., Van Der Windt D.A.W.M., Stalman W.A.B. & Verhagen A.P., 2007, ‘Survey among 78 studies showed that Lasagna’s Law holds in Dutch primary care research’, *Journal of Clinical Epidemiology* 60(8), 819–824. 10.1016/j.jclinepi.2006.11.010 17606178

[CIT0033] Willms J.D., 2003, *Student engagement at school: A sense of belonging and participation*, viewed 13 July 2018, from https://www.oecd.org/education/school/programmeforinternationalstudentassessmentpisa/33689437.pdf

[CIT0034] World Health Organization, 1997, *Promoting health through schools*, viewed 13 July 2018, from http://whqlibdoc.who.int/trs/WHO_TRS_870.pdf

[CIT0035] World Health Organization, 1998a, *Health-promoting schools: A healthy setting for living, learning and working*, viewed 13 July 2018, from http://apps.who.int/iris/bitstream/10665/63868/1/WHO_HPR_HEP_98.4.pdf

[CIT0036] World Health Organization, 1998b, *Healthy nutrition: An essential element of a health-promoting school*, viewed 13 July 2018, from http://apps.who.int/iris/bitstream/handle/10665/63907/WHO_HPR_HEP_98.3.pdf

[CIT0037] World Health Organization, 2012, *Landscape analysis on countries’ readiness to accelerate action in nutrition*, viewed 13 July 2018, from http://www.who.int/nutrition/landscape_analysis/en/

[CIT0038] Worldatlas, 2017, *What is the capital of South Africa?*, viewed 13 July 2018, from https://www.worldatlas.com/articles/what-is-the-capital-of-south-africa.html

